# Supplementary material for: Umbelliferose Isolated from Cuminum cyminum L. Seeds Inhibits Antigen-Induced Degranulation in Rat Basophilic Leukemia RBL-2H3 Cells
Source: Molecules. 2022 Jun 25;27(13):4101. doi: 10.3390/molecules27134101 (PMC9268690; doi:10.3390/molecules27134101)
Supplement: Supplementary file 1 [file molecules-27-04101-s001.zip › molecules-1780122-supplementary.pdf]

Figure S1. NMR spectrum of umbelliferose

	^{1}H-NMR	^{13}C-NMR	H_2BC	HMBC	TOCSY	^{1}H-^{1}H COSY
1	δ 3.66 (*m*)	δ 61.8	-	-	-	-
2	-	δ 104.3	-	-	-	-
3	δ 4.13 (*d*, *J* = 8.6 Hz)	δ 76.2	δ 73.7	δ 61.8, 73.7	δ 3.76, 4.02	δ 4.02
4	δ 4.02 (*dd*, *J* = 8.6, 8.6 Hz)	δ 73.7	δ 76.2, 81.1	δ 62.0, 76.2, 81.0	δ 3.76	δ 3.76
5	δ 3.76 (*m*)	δ 81.1	δ 73.7	δ 73.7	-	δ 3.71, 4.02
6	δ 3.71, 3.75 (*m*)	δ 62.0	-	δ 73.7 (δ 3.71) δ 81.1 (δ 3.71)	-	-
1'	δ 5.60 (*d*, *J* = 3.6 Hz)	δ 89.5	δ 75.5	δ 71.2, 72.1, 75.5, 104.3	δ 3.43, 3.59, 3.80	δ 3.59
2'	δ 3.59 (*dd*, *J* = 3.6, 9.9 Hz)	δ 75.5	δ 71.2	δ 71.2, 96.4	δ 3.43-	-
3'	δ 3.80 (*m*)	δ 71.2	δ 75.5	δ 69.2, 75.5	δ 3.43, 3.59	δ 3.59
4'	δ 3.43 (*dd*, *J* = 9.6, 9.6 Hz)	δ 69.2	δ 72.1	δ 60.2, 71.2, 72.1	δ 3.59	δ 3.85
5'	δ 3.85(*m*)	δ 72.1	δ 69.2	-	δ 3.43, 3.59, 3.72	δ 3.43
6'	δ 3.72, 3.74 (*m*)	δ 60.2	-	δ 69.2 (δ 3.72)	δ 3.43 (δ 3.74) δ 3.59 (δ 3.74)	-
1"	δ 5.08 (*d*, *J* = 3.9 Hz)	δ 96.4	δ 68.1	δ 69.3, 71.0, 75.5	δ 3.82, 3.93	δ 3.77
2"	δ 3.77 (*m*)	δ 68.1	δ 69.3	-	-	-
3"	δ 3.82 (*dd*, *J* = 3.2, 10.5 Hz)	δ 69.3	δ 68.1	δ 68.1	-	δ 3.77
4"	δ 3.93 (*d*, *J* = 3.2 Hz)	δ 69.0	-	δ 68.1, 69.3	δ 3.82	-
5"	δ 4.08 (*t*, *J* = 6.3 Hz)	δ 71.0	δ 61.0	δ 61.0, 69.0, 96.4	δ 3.65	δ 3.65
6"	δ 3.65 (*m*)	δ 61.0	δ 71.0	δ 69.0, 71.0	-	δ 4.08

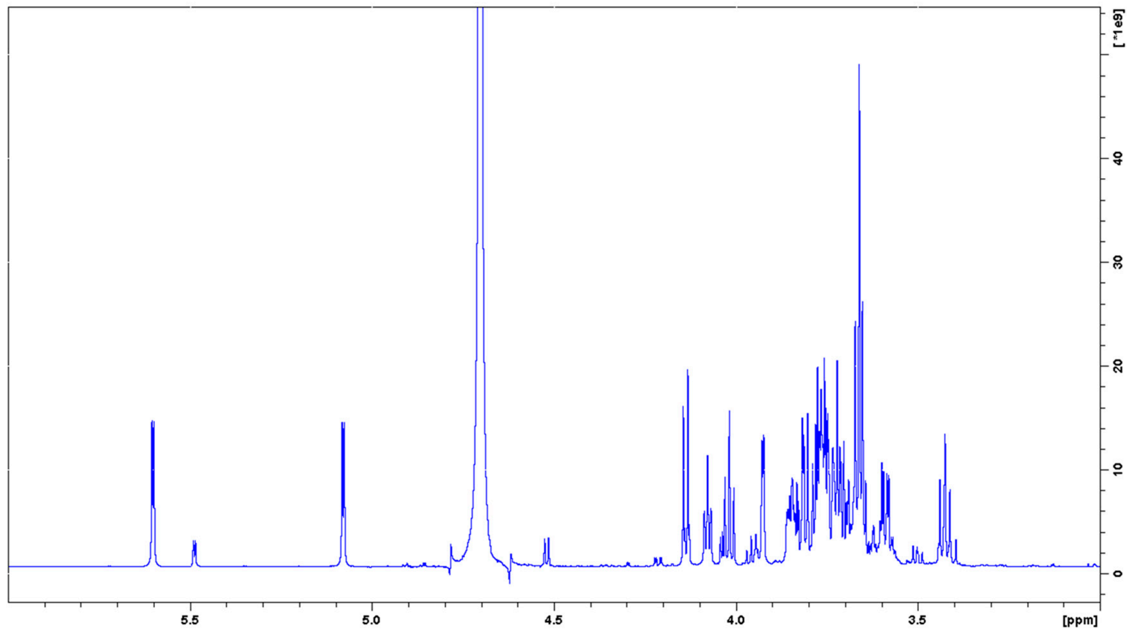

[1] H-NMR spectrum (700 MHz, D_2O) of umbelliferose

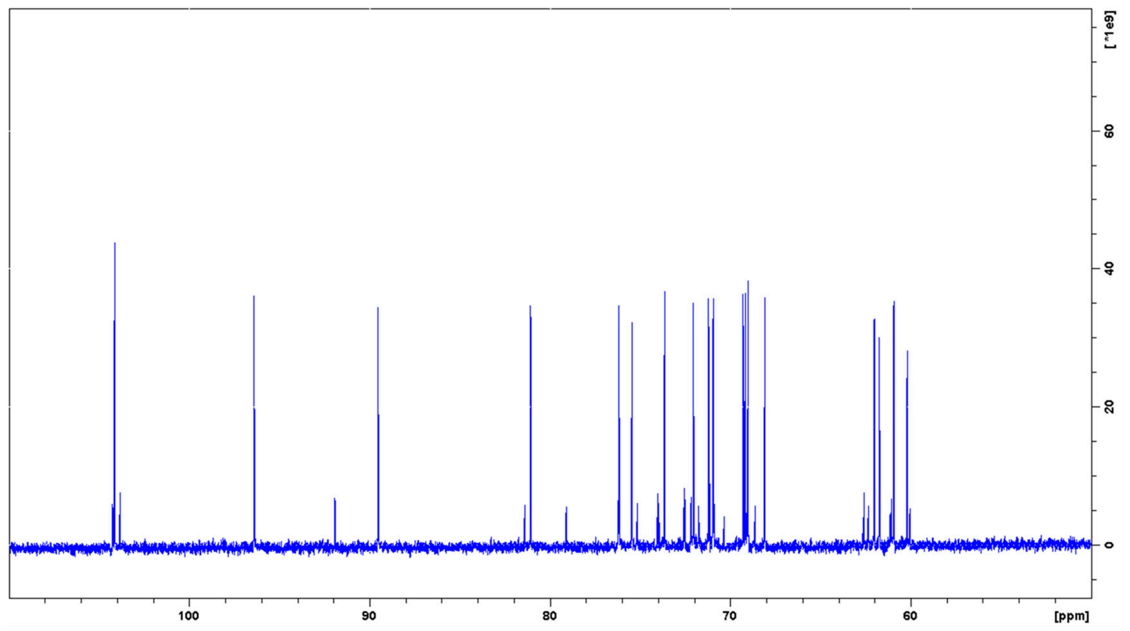

^{13}C-NMR spectrum (175 MHz, D_2O) of umbelliferose

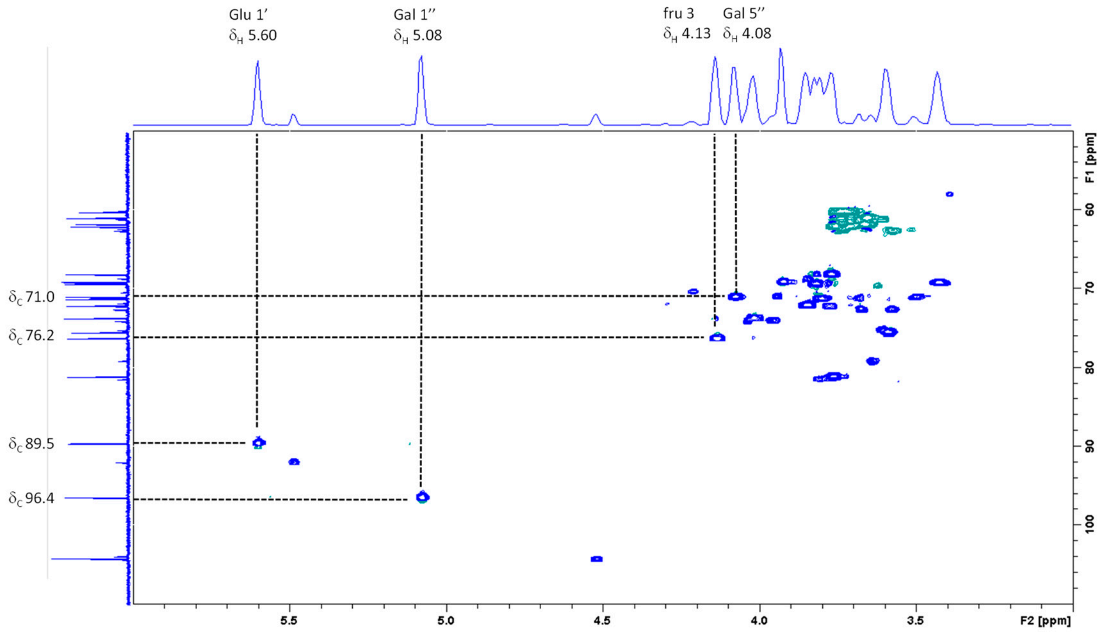

HSQC spectrum (700 MHz, D_2O) of umbelliferose

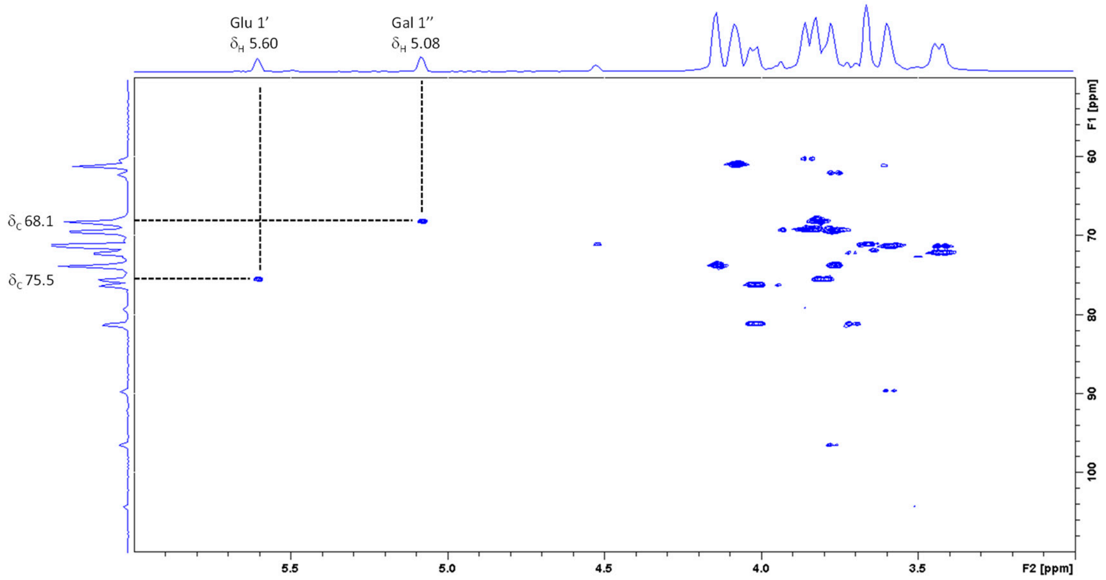

H_2BC spectrum (700 MHz, D_2O) of umbelliferose

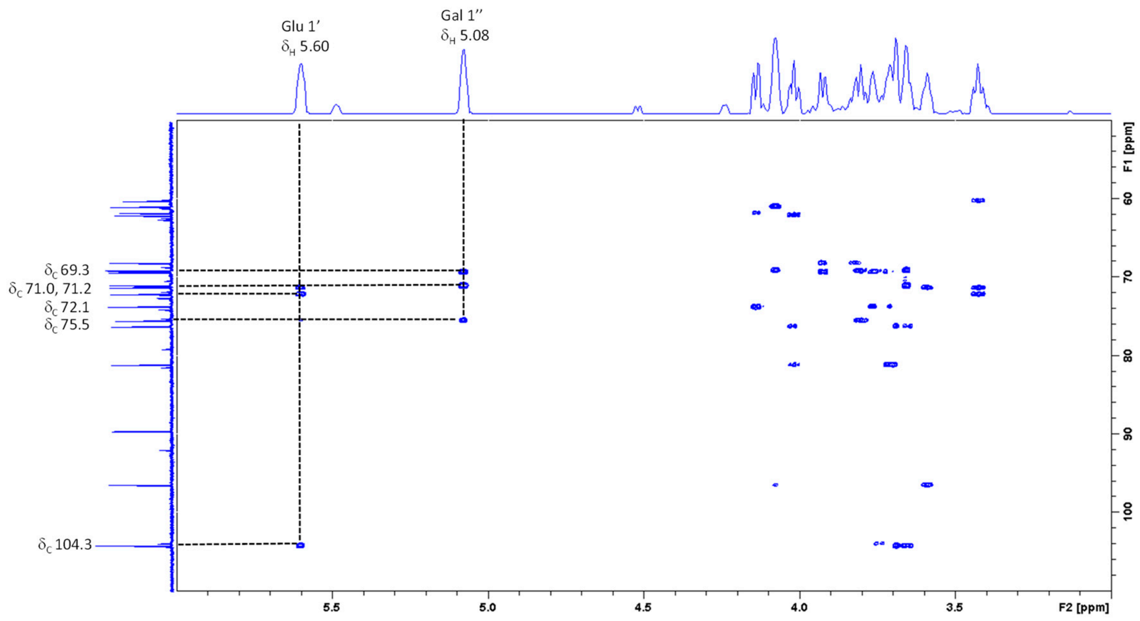

HMBC spectrum (700 MHz, D_2O) of umbelliferose

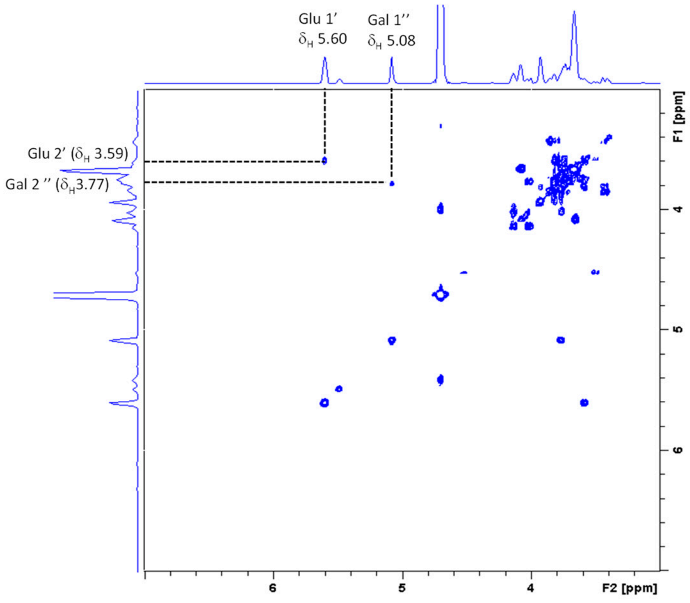

^1H-^1HNMR COSY spectrum (700 MHz, D_2O) of umbelliferose

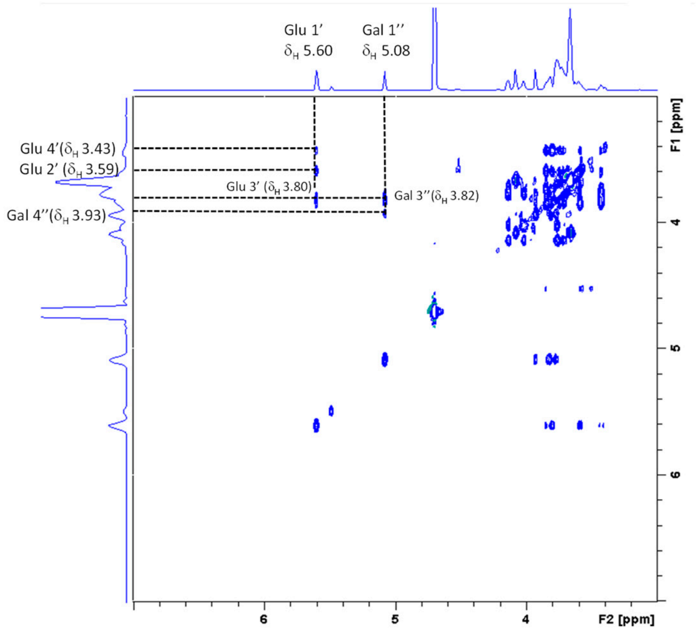

TOCSY spectrum (700 MHz, D_2O) of umbelliferose
